# Towards the Immunoproteome of *Neisseria meningitidis*

**DOI:** 10.1371/journal.pone.0005940

**Published:** 2009-06-16

**Authors:** Tom A. Mendum, Jane Newcombe, Celia L. McNeilly, Johnjoe McFadden

**Affiliations:** Faculty of Health and Medical Sciences, University of Surrey, Guildford, United Kingdom; Federal University of São Paulo, Brazil

## Abstract

Despite the introduction of conjugated polysaccharide vaccines for many of the *Neisseria meningitidis* serogroups, neisserial infections continue to cause septicaemia and meningitis across the world. This is in part due to the difficulties in developing a, cross-protective vaccine that is effective against all serogroups, including serogroup B meningococci. Although convalescent *N. meningitidis* patients develop a natural long-lasting cross-protective immunity, the antigens that mediate this response remain unknown. To help define the target of this protective immunity we identified the proteins recognized by IgG in sera from meningococcal patients by a combination of 2D protein gels, western blots and mass spectrometry. Although a number of outer membrane antigens were identified the majority of the antigens were cytoplasmic, with roles in cellular processes and metabolism. When recombinant proteins were expressed and used to raise sera in mice, none of the antigens elicited a positive SBA result, however flow cytometry did demonstrate that some, including the ribosomal protein, RplY were localised to the neisserial cell surface.

## Introduction

*Neisseria meningitidis* infection continues to cause considerable disability and mortality throughout the world. Although polysaccharide conjugate vaccines have been developed and used successfully against many of the serogroups of *N. meningitidis*, such a strategy has proved ineffective against group B meningococci. This serogroup now represents the majority of cases in the UK [1]. Vaccines containing Outer Membrane Vesicle preparations (OMVs) have been used to successfully to vaccinate against specific outbreak strains of group B *N. meningitidis* [2], [3], [4], but such preparations do not give sufficient cross protection to justify their use as universal meningococcal vaccines. Although protein antigens that are protective against serogroup B meningococci have been identified by reverse vaccinology [5] there remains a need to characterise the immune response to neisserial infection and to identify further vaccine candidates for use in a cross protective vaccine.

Classic studies performed in the 1960's demonstrated the importance of bactericidal antibody for protection against meningococcal disease, as reviewed by Pollard and Frasch [6]. Several lines of evidence suggest that important and pan-reactive determinants of immunity remain to be discovered. (i) In immunocompetent individuals a single episode of meningococcaemia confers permanent immunity to all types of meningococci [6], [7], [8]. (ii) Carriage of commensal species such as *Neisseria lactamica* provides immunity to meningococcal disease [6], [7], [8]. (iii) Inoculation of mice with attenuated mutant meningococcal strains induces cross-reactive immune responses [9]. These findings indicate that natural exposure (to either *N. meningitidis* or *N. lactamica*) can provide long-term, cross-reactive protection, however the identity of the antigens involved remains unknown. They are unlikely to be the well characterised class 1, 2 and 5 Outer Membrane Proteins (OMPs) since these antigens do not induce a cross-reactive bactericidal immune responses in immunized volunteers [10].

The proteome of both the serogroup B strain, MC58 [11], and the serogroup A strain, Z4970 [12], as well as the composition of OMV preparations from both *N. meningitidis* [13], [14], [15], [16], [17] and *N. lactamica* [13], [14], [17] have been catalogued using a combination of 2D SDS-PAGE gels and mass spectrometry (reviewed by Wheeler *et al.* [18]). However, the proteome itself does not provide information as to which proteins are immunogenic. By combining proteomics with immunoblotting it is possible to generate an immunoproteome that catalogues those proteins that are recognized by the host immune response. This immunoproteomics approach has been applied to a wide of range of organisms including *M. tuberculosis* [19], *Streptococcus pneumonia* [20], *Staphylococcus epidermidis* [21] and *Candida albicans* [22]. In many cases this has led to the identification of novel antigens that have been demonstrated to be protective in animal models [20], [21], [22]. We here, apply immunoproteomic approaches to *N. meningitidis* to identify proteins that bind IgG from acute and convalescent meningococcal patient's sera with the aim of further understanding the immune response to neisserial infection and to potentially identify new cross-protective neisserial antigens.

## Results

### 2D electrophoresis and Western blots

**Figure 1:**
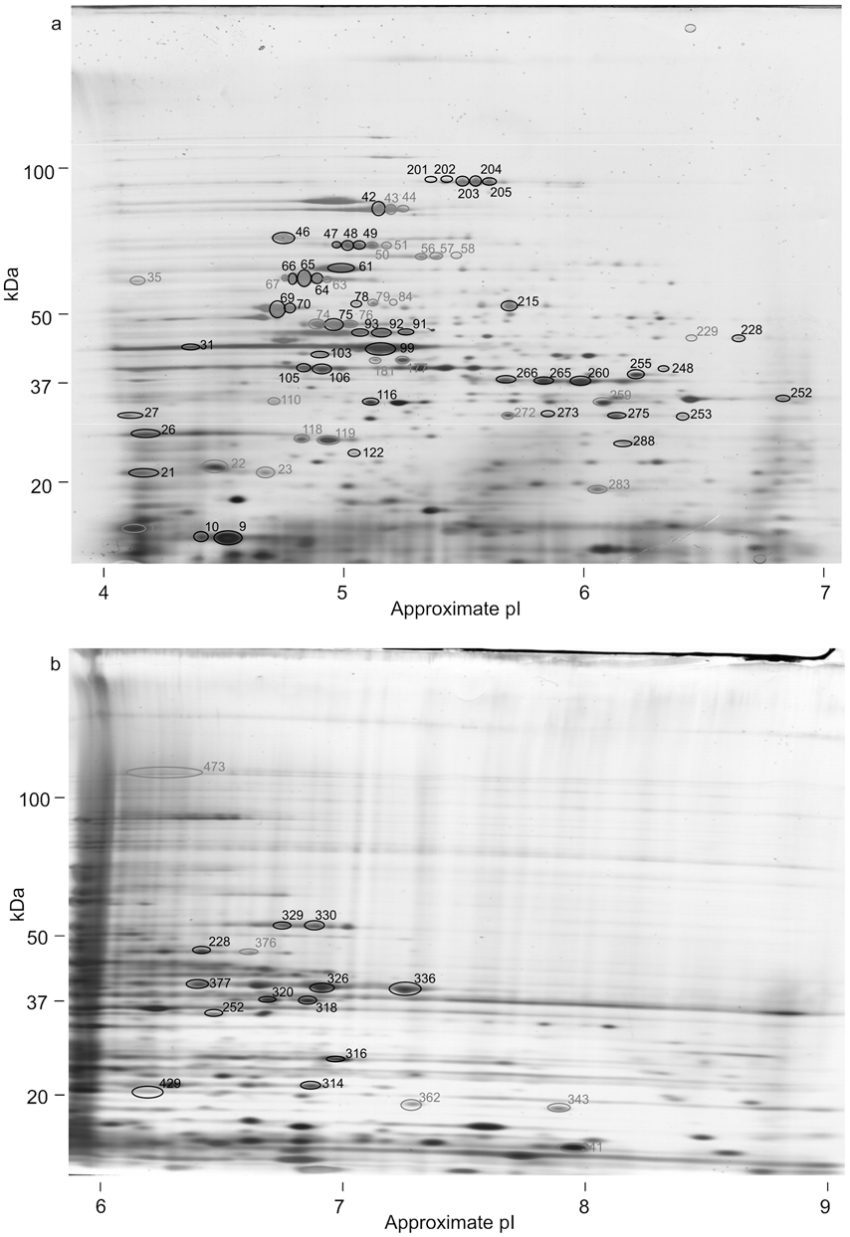
2D gels of total *N. meningitidis* proteins. Total *N. meningitidis* proteins separated by 2D gel electrophoresis using (a) a non-linear pI 4–7 1^st^ dimension and (b) a non-linear pI 6–9 1^st^ dimension. Gels were silver stained and replica gels western blotted with patient sera. Spots that were recognised by one or more sera on western blots are circled. Spots whose identity was determined are numbered in black, those that remain unidentified in grey.

**Figure 2:**
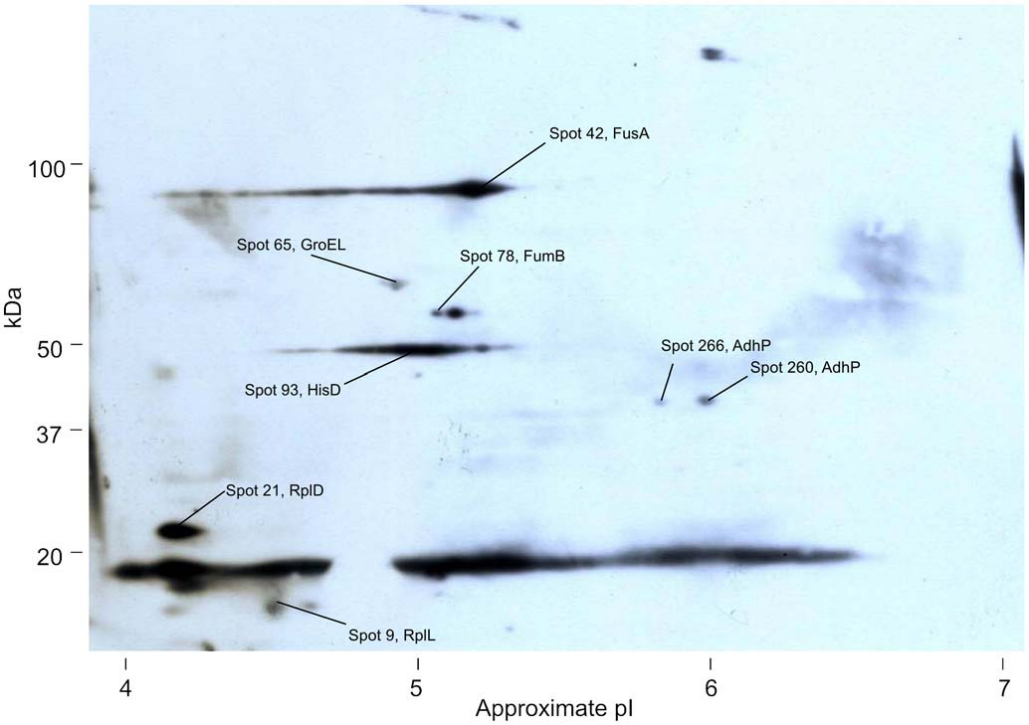
An example western blot of a 2D gel of total protein. Total *N. meningitidis* proteins separated by 2D gel electrophoresis using a non-linear pI 4–7 1^st^ dimension, western blotted, and probed with convalescent sera from patient 3. Spots that were assigned protein identities are indicated. Spots with no protein legend have not been identified. Longer exposures of this blot revealed more spots (Table 2).

Proteins extracted from *N. meningitidis* L91543 were separated by 2D gel electrophoresis and western blotted with sera from acute and convalescent patients (Table 1). Up to 473 separate protein spots could be distinguished on the 2D gels (Fig 1). Eighty eight of these 473 spots bound sufficient IgG from one or more of the patient sera to be detected on western blots (Fig 2).

**Table 1:** Details of patient sera.

Patient	Strain	Patient History
1	C:NT:P1.5,2	Severe septicaemia
2	nd^a^	Mild septicaemia
3	B	Not known
4	nd^a^	Severe septicaemia
5	B	Severe septicaemia
6	B	Mild septicaemia
7	B:4:P1.4	Not known
8	B:4:NT	Not known
9	B:4:NT	Not known
10	B:NT:P1.9	Not known
11	B:4:P1.4	Not known
12	B:4:P1.4	Not known
13	B:15:P1.7,16	Not known
14	B:4:P1.4	Not known
15	B:4:P1.4	Not known
16	B:4:P1.4	Not known
17	C	Not known
18	C	Not known
19	C	Not known
20	W135	Not known
21	Y	Not known

Comparison of western blots immunoprobed with different patient sera demonstrated clear differences in immune recognition with individual patient serum recognising between one and 41 protein spots (Table 2). Many proteins were recognized by several sera while others were specific to a single serum sample. As expected, all the acute sera bound to fewer spots than their paired convalescent sera. Unfortunately there was insufficient patient information to attempt to relate immunogenic profiles to patient details. No correlation to the infecting strain serotypes was apparent, nor was there any obvious correlation to SBA titres (most of the acute sera had high levels of complement independent killing so an SBA titre could not be obtained). Replicate western blots identified similar spot patterns.

**Table 2:** Identities of 2D gel spots that bind IgG from acute (Ac) and convalescent (Con) sera.

Patient				1	1	2	2	3	3	4	4	5	6	7–11	12–16	17–22
				Ac	Con	Ac	Con	Ac	Con	Ac	Con	Con	Con	Con	Con	Con
SBA titers (L91543)^a^				2	>512	nt	>512	nt	128	nt	32	>512	>512	32	>512	>512
Spot	Protein	Name	NMB													
9	RplL	50S ribosomal protein L7/12	0131	Y	Y	Y	Y	Y	Y	Y	Y					
10	Hypo	Hypothetical	2015						Y		Y					
21	RplD	50S ribosomal protein L4	0143			Y	Y		Y							Y
26	n/a	Opa900	n/a		Y				Y							
27	RmpM	OMP class 4	0382					Y	Y							
31	TufA	Elongation factor Tu	0139		Y				Y							
42	FusA	Elongation factor G	0138			Y	Y	Y	Y		Y			Y		Y
46	DnaK	DnaK protein (hsp)	0554	Y	Y											
47	LpdA	OMP p64k	1344					Y	Y							Y^b^
48	LpdA	OMP p64k	1344					Y	Y							Y^b^
49	LpdA	OMP p64k	1344			Y		Y	Y							Y^b^
61	RpsA	30S ribosomal protein S1	1301				Y									
64	GroEL	Hsp60	1972		Y				Y							Y^b^
65	GroEL	Hsp60	1972		Y	Y	Y		Y	Y	Y					Y^b^
66	GroEL	Hsp60	1972		Y				Y							Y^b^
69	Pta	Phosphate acetyltransferase	0631						Y							Y^b^
70	Pta	Phosphate acetyltransferase	0631						Y							Y^b^
75	AtpD	ATP synthase F1 beta chain	1934		Y				Y							
78	FumB	Fumarate hydratase	1613				Y		Y					Y		
91	SfcA	Malate oxidoreductase	0671								Y					
92	SfcA	Malate oxidoreductase	0671								Y					
93	HisD	Histidinol dehydrogenase	1581		Y		Y	Y	Y							Y
99	TufA	Elongation factor Tu	0139		Y											Y
103	FtsZ	Cell division protein	0427						Y							
105	SucC	Succinyl coA synthase	0959		Y											
106	SucC	Succinyl coA synthase	0959		Y											
116	Tsf	Elongation Factor Ts	2002						Y							
122	Adk	Adenylate kinase	0823						Y							
201	AcnB	Aconitate hydratase 2	1572						Y							
202	AcnB	Aconitate hydratase 2	1572		Y				Y							
203	AcnB	Aconitate hydratase 2	1572		Y				Y							
204	AcnB	Aconitate hydratase 2	1572		Y											
204	AcnB	Aconitate hydratase 2	1572		Y											
205	AcnB	Aconitate hydratase 2	1572		Y											
248	PilT-2	Pilin retraction protein	0768													Y
252	CysK	Cysteine synthase	0763						Y							
253	RmpM	OMP class 4	0382													Y
255	GcvT	Glycine cleavage system T protein	0574				Y									
260	AdhP	Alcohol dehyrogenase	0546				Y									
265	AdhP	Alcohol dehyrogenase	0546				Y									Y
266	AdhP	Alcohol dehyrogenase	0546			Y	Y									
273	RmpM	OMP class 4	0382						Y							Y^b^
275	RmpM	OMP class 4	0382						Y							Y^b^
288	FabI	Enoyl-reductase	0336			Y	Y									
314	RplY	Ribosomal protein L25	0876				Y									Y
316	EtfB	Flavoprotein, beta subunit	2155		Y							Y	Y			
318	PilT-1	Pilin retraction protein	0052									Y		Y		Y
320	RfaC	Heptosyltransferase I	2156											Y		Y
320	NagZ	Glycosyl hydrolase	0530						Y							
326	PorA	OMP PorA	1429											Y	Y	
329	GuaB	IMP dehydrogenase	1201		Y											
330	GuaB	IMP dehydrogenase	1201		Y											
336	PorA	OMP PorA	1429												Y	
377	PorA	OMP PorA	1429												Y	
Unidentified proteins as referred to in Fig. 1					12	50	79	22	22			229		79		43
					35	79		50	23			341		84		44
					74			51	35					473		50
								56	50							63
								57	51							67
								58	56							79
								61	57							110
								177	58							118
									79							119
									177							229
									181							272
									283							343
																362
																376

a nt – No titer. Some of the SBA assays of acute sera showed high levels of complement independent killing. This is possibly associated with the administration of antibiotics to the patients.

b – Antigens that were also identified from western blots of OMV preparations.

Although 2D gels of OMVs clearly had different protein profiles to gels with total protein preparations, no novel antigens were identified when they were western blotted with the three mixed convalescent sera (Table 2).

### Identification of antigens

Immunogenic proteins were excised from the gels and identified by mass spectrometry. Of the 88 spots that were detected on the western blots, 54 were successfully identified by mass spectrometry, representing 33 different proteins (Table 2, Table S1). Many of the proteins were identified from more than one spot, often with the same molecular weight but with differing pI values. Presumably, these represent isoforms of the same protein that retain their immunogenicity. Some of these isoforms appear to be immunologically distinct from each other. For instance spots 105 and 106, both identified as SucC were recognized by IgG in convalescent sera from patient 1, however the spot adjacent to 106 that was also identified as SucC, but did not bind IgG (Fig 1).

The immunogenic proteins identified (Table 2) represent a wide range of functions, including chaperones, ribosomal proteins and many that are involved in central metabolism. Most of these proteins are predicted to be cytoplasmic and therefore are not expected to be directly accessible to the immune system in intact cells. This was despite the presence of many of the established outer membrane antigenic proteins such as PorA, PorB, Opa, and RmpM on the 2D gels.

### Cloning, Expression and Confirmation of antigens

Genes encoding the protein antigens were PCR amplified and cloned into the expression vector, pET101. Eighty two percent of the proteins were successfully expressed. Most of these recombinant proteins were demonstrated, by western blotting, to bind the patient sera with which they were originally identified (Table 3), confirming that the mass spectrometry identification was correct. Of those tested only the putative nucleotidase NMB2015, 50S ribosomal protein L4 (NMB0143) and a putative phosphate acetyl transferase, NMB0631, did not bind the original patient sera indicating that either the wrong spot was picked from the 2D gels, that the protein was mis-identified or that the recombinant protein has lost the epitopes that were present on the native protein.

**Table 3:** Proteins that bound IgG from one or more of the patients sera.

NMB	Name	Protein	Confirmed^a^	Literature
0052	Pilin retraction protein	PilT-1	nd	
0131	50S ribosomal protein L7/L12	RplL	Y	Evidence for a surface role in *N. gonorrhoeae* during cell invasion [23], [24]
				Immunoreactive in *Chlamydia trachomatis* [25]
				Protective against *Brucella abortus* in mouse model [26]
				Neisserial L7/12 shown to be immunogenic but with limited immunogenicity for T cells [27]
				Identified on the surface of *Streptococcus oralis* [28]
0138	Elongation factor G	FusA	Y	*Thermus thermophilus* elongation factor G shown to be putatively membrane associated [29]
				Proposed as a diagnostic antigen in *Helicobacter pylori* [30].
				Identified on the surface of *Streptococcus oralis* [28]
0139	Elongation factor Tu	TufA	Y	Fibronectin binding in *Mycoplasma pneumoniae* [31]
				Cell wall associated in *Mycobacteria leprae* [32]
				Surface associated and associated with cell adhesion in *Lactobacillus johnsonii* [33]
				Bovine IgG binds Ef-Tu on surface of *Anaplasma marginale* [34]
				Surface located virulence factor on *Pseudomonas aeruginosa*, binding factor H and plasminogen [35]
				Immunoreactive in *Chlamydia trachomatis* [25].
0143	50S ribosomal protein L4	RplD	N	
0336	Enoyl reductase	FabI	Y	
0382	OMP class 4	RmpM	Y	Well documented surface protein [36].
0427	FtsZ	FtsZ	Y	Immunogen in convalescent *Borrelia burgdorferi* patients [37]
				A structural homolog is antigenic in *Bartonella bacilliformis* [38]
0530	Beta-hexosaminidase	NagZ	nd	
0546	Alcohol dehyrogenase	AdhP	nd	Immuno-reactive in patients with systemic candidiasis [39]
0554	DnaK protein (hsp)	DnaK	nd	Immunoreactive and partially protective in mouse model for *Bruclla abortus* [40]
				Localized to the surface in *Neisseria meningitidis* [41]
				Immunoreactive in *Chlamydia trachomatis* [25].
0574	Glycine cleavage system T protein	GcvT	Y	
0631	Phosphate acetyltransferase	Pta	N	
0671	Malate oxidoreductase	SfcA	Y	Found in the immunoproteome of *Brucella abortus* [42]
				Putative serodiagnostic with *Mycobacterium tuberculosis* [43]
0763	Cysteine synthase	CysK	Y	Found in the immunoproteome of *Brucella abortus* [42]
0768	Pilin retraction protein	PilT-2	Y	
0823	Adenylate kinase	Adk	Y	Identified on the surface of *Streptococcus oralis* [28]
0876	Ribosomal protein L25	RplY	Y	Antigens found in patients with borreliosis [44]
0959	Succinyl coA synthase	SucC	Y	
1201	IMP dehydrogenase	GuaB	Y	Immunoreactive and protective in mice infected with *Candida albicans* [22]
1301	30S ribosomal protein S1	RpsA	Y	Immunoreactive in *Chlamydia trachomatis* [25]
1344	OMP p64k, PDH, E3 component	LpdA2	Y	Outer membrane location in *Neisseria* [45]
				Recognised by sera from convalescent neisserial patients [46]
1429	OMP PorA	PorA	nd	Well documented OMP
1572	Aconitate hydratase 2	AcnB	Y	Immuno-reactive in patients with systemic candidiasis [39]
1581	Histidinol dehydrogenase	HisD	Y	
1613	Fumarate hydrytase	FumB	nd	Found in the immunoproteome of *Brucella abortus* [42]
1934	ATP synthase F1 beta chain	AtpD	Y	
1972	Hsp60	GroEL	Y	Conserved proteins that are immunogenic in many bacterial pathogens
2002	Elongation Factor Ts	Tsf	nd	Identified on the surface of *Streptococcus oralis* [28]
2015	Hypothetical	-	N	
2155	e transfer flavoprotein, beta subunit	EtfB	nd	
2156	Heptosyltransferase I	RfaC	nd	
n/a	Opa900(FAM18), not in MC58	n/a	nd	Well documented OMP

a Recombinant proteins that were confirmed as binding their identifying sera are indicated (Y), those that failed to bind their identifying sera are indicated (N) and those that were not determined, either because no recombinant protein was generated or because no more sera was available are indicated (nd).

### ELISAs with recombinant proteins and cell lysates

Recombinant proteins were used to raise sera in mice. All of the sera gave ELISA titers of >1∶10,000 against recombinant protein except for FtsZ which had a titer of 1∶80. In ELISAs with cells that had been heat killed and sonicated, all of the sera except for FtsZ, PilT-2, Adk, AcnB and HisD recognized antigens from the meningococcal cells, indicating that they were present in amounts and in a conformation that could be recognized by the sera raised against the recombinant protein.

### Serum Bactericidal Assays

None of the successfully expressed antigens identified in this study gave a positive SBA of ≥1∶4. Control assays using OMVs and recombinant fHBP consistently gave titres >1∶512. No killing was seen in control wells lacking either active complement, sera, or both.

### Flow cytometry

**Figure 3:**
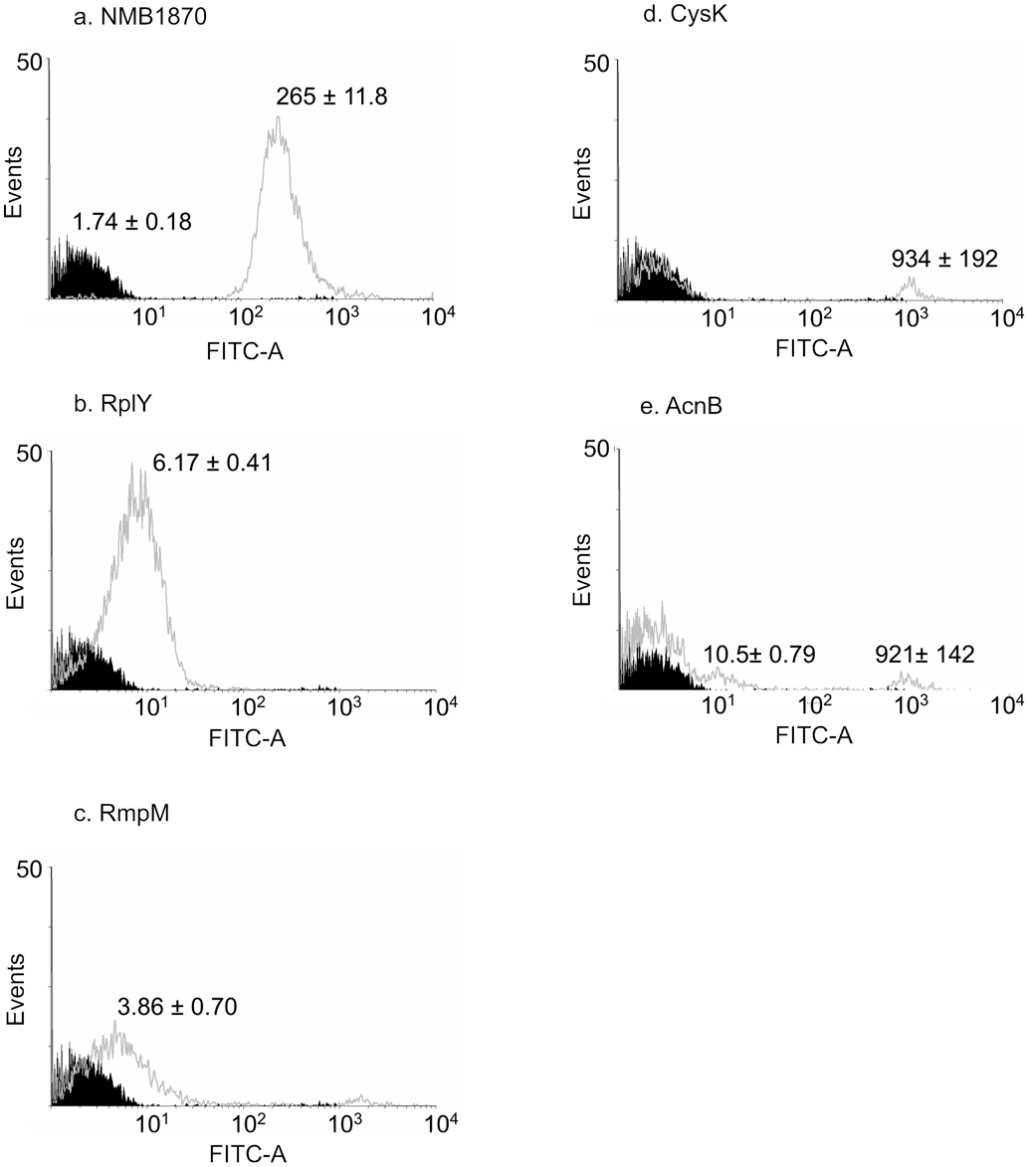
Flow cytometry of live *N. meningitidis* stained with mouse sera immunised with recombinant proteins. Flow cytometry data for live *N. meningitidis* cells stained with sera from mice immunised with PBS alone (black) or with recombinant proteins (grey), fHBP has been used as a positive control (Fig 3a). Values are means of fluorescence with standard deviations derived from triplicate experiments.

The subcellular location of antigenic proteins was investigated by flow cytometry. Sera raised against the meningococcal surface protein, fHBP was used as a positive control (Fig 3a), while sera raised in mice immunised with PBS was used as a negative control. Several of the recombinant proteins raised IgG that bound to the surface of whole, live cells. RplY was clearly available on the surface, with a significant shift in the fluorescence of the entire population (p = 0.001) (Fig 3b). Sera raised against RmpM, CysK and AcnB also showed small but significant shifts in fluorescence of the total population as well as a small but significant subpopulation of cells with relatively high levels of fluorescence (Fig 3). The subpopulations with high fluorescence had similar side scatter and forward scatter profiles to the whole population indicating that the cells were intact.

## Discussion

Our aim in this study was to begin to characterize the meningococcal immunoproteome and possibly to identify novel antigens that are the targets of natural cross-protective immunity. By using a combination of 2D gels, western blots and proteomics, we identified a set of proteins that were recognized by sera from both acute and convalescent patients suffering from meningococcal septicaemia. This approach contrasts with previous studies aimed at identifying naturally occurring immunological responses such as Litt *et al.* [47], in that preconceived criteria, such as *in silico* predications of protein function, were not applied.

Both the number and identity of the proteins identified by each sera varied considerable. No antigen was recognized by all the sera and several antigens were recognised by only a small number of patient serum samples. The origin of this diversity of immune response is currently unknown. It is possible it represents innate differences in immunological responsiveness to the antigens, or variations in antigenic exposure in different patients due to variations in treatment timings or disease severity.

The antigens that were identified are a diverse group of proteins, ranging from known outer membrane proteins such as RmpM, to enzymes of central metabolism and ribosomal proteins (Table 2). Where possible the immunogenicity of the identified proteins was confirmed by cloning, protein expression and by demonstrating that the recombinant proteins were recognized by patient sera (Table 3). Many of the proteins are predicted to be located in the cytoplasm or inner membrane of the meningococcus and so were not considered by Litt *et al.* [47] in their screen of immunological responses to infection. Other studies that have used similar 2D gel/western blot approaches to identify antigens have been undertaken using a range of organisms including *Helicobacter pylori* [30] *Chlamydia trachomatis* [25], *Borrelia burgdorferi* [37], and *Mycobacteria tuberculosis* [19]. These have also generated lists of antigenic proteins that contain large numbers of proteins with a cytosolic function. For instance, DnaK, CysK, FumB and AtpA have all been identified from *Brucella abortus* as immunoreactive by Connolly *et al.* [42], while TufA, DnaK and RpsA have been identified as immunoreactive in *Chlamydia trachomatis* [25]. The most obvious explanation for these immune responses is that they are generated during episodes of septicaemia when killed meningococcal cells release their cytoplasmic contents into the circulation. Such a scenario would suggest that although these antigens may be involved in the immune response to septicaemic meningococcal disease, they are unlikely to be good vaccine candidates.

However, we have demonstrated that several of these “cytoplasmic” proteins were present on the surface of neisserial cells and so may be involved in immune responses that are relevant to live meningococcal cells. The presence of neisserial “cytoplasmic” proteins on the surface has been reported previously, for example Ferrari *et al.* [48] found enolase, Hsp60, TufA and glyceraldehyde 3-phosphate dehydrogenase on the neisserial surface by flow cytometry while other researchers have identified RplL [23], [24], DnaK [41], and LpdA2 [45] as being surface exposed. The flow cytometry data presented here demonstrates that the ribosomal protein L25 (RplY) is available at the cell surface with levels of fluorescence comparable to those found with recognised surface antigens such as NMB1468 [49], NMB2132 and NMB2091 [5], although considerably lower than other surface antigens such as fHBP [5]. A different pattern of surface localization was found for CysK and AcnB, were flow cytometry indicated that the proteins were surface exposed at relatively high amounts but only in a subpopulation of cells. Similar results were obtained in these experiments with RmpM, a known outer membrane protein. Such heterogeneity in surface expression has been found with several proteins in *Streptococcus pneumoniae* and has been attributed to variation in capsule morphology [50]. As we used a capsulated strain it is possible that variations in meningococcal capsule morphology may similarly expose meningococcal antigens to recognition by the host immune response in a proportion of the neisserial population. Alternatively, surface exposure of these proteins may be transient, only occurring during specific phase of the meningococcal cell cycle, or be subject to phase variation.

None of the identified immunogenic proteins generated murine immune sera with bactericidal activity. For those proteins not expressed on the surface it is likely that the absence of SBA activity is due to lack of accessibility of antibody to the antigens. For those proteins with either low or transient surface expression it seems likely that either the level of antigen on the surface is insufficient to promote bactericidal killing or, for transiently expressed proteins, only a fraction of the population may be killed. Such proteins would be missed by the current bactericidal assays.

These data represent the first proteome wide investigation of the naturally induced immune response to neisserial infection with implications for the understanding of the immune response to septicaemic infection. The antigens identified demonstrate that meningococcal patients have highly variable immune responses against a wide range of meningococcal antigens. All the sera tested contain antibodies capable of binding a range of neisserial proteins, including many “cytoplasmic” proteins, some of which are for the first time shown to be available to IgG on the surface of whole neisserial cells. Although not sufficient to promote detectable complement mediated killing, immune responses against these antigens may still be involved in the pathogenesis and immunity to meningococcal disease.

## Materials and Methods

### Protein preparation

*Neisseria meningitidis* L91543 was used thoughout (obtained from the Manchester Public Health Laboratory, UK), this is a group C:2a:P1.2, and part of the ST-11, ET-37 complexes. Although such a serogroup C strain is not suited to the identification of vaccine antigens aimed at serogroup B *Neisseria*, it is applicable for the identification of the cross reactive antigens which this study aimed to discover. Protein for 2D gels was prepared from cultures grown for 4 h on Columbia Agar Base supplemented with 6% horse blood (CAB) at 37°C with 5% CO_2_. Cells were washed in PBS and proteins prepared using a ProteoPrep® Sample Extraction Kit (Sigma). Cells were resuspended in Reagent 4 and sonicated for 10 min. The cells were centrifuged for 5 min and the pellet discarded, freshly prepared Tributylphosphine (TBP) was added to the supernatant, and the mixture incubated for 1 h at room temperature. The proteins were alkylated with iodoacetamide (IAA) for 1.5 h at room temperature. OMVs were prepared using sodium deoxycholate from 4 h old CAB plate cultures according to Fredriksen *et al.* [51].

### 2D gel electrophoresis

Protein concentrations were determined using *RC DC* Protein Assay (BioRad). Zoom® 1^st^ dimension strips (Invitrogen) were rehydrated for 18 h with 20 µg protein in 155 µl rehydration buffer (8 M urea, 2% CHAPS, 50 mM dithiothreitol (DTT), 0.5% Zoom® carrier ampholytes of an appropriate pI range (GE Healthcare), 0.002% bromophenol blue) and focused at 200 V for 15 min, 450 V for 15 min, 750 V for 15 min followed 2000 V for 140 min. Strips were alkylated with 1% DTT in equilibration buffer (1.5 M Tris, 6 M urea, 30% glycerol, 2% SDS, 0.002% bromophenol blue, pH 8.8) for 15 min, and then reduced with 4% IAA in equilibration buffer for 15 min. Focused 1^st^ dimension strips were loaded onto 2^nd^ dimension NuPAGE® Novex 4–12% Bis-Tris ZOOM® gels (Invitrogen) and electrophoresed at 200 V as recommended by the manufacturers. Gels were silver stained as described by Mortz *et al.* [52].

### Serum

Four pairs of acute and convalescent sera (patients 1, 2, 3 and 4); two single convalescent serum (patients 5 and 6) and three samples of pooled serum, each containing five convalescent sera (patients 7–11, 12–16, and 17–21), were used to probe 2D gels of total neisserial proteins (Table 1). OMV preparations were only probed with the three convalescent sera mixes.

### Western blots

Replica 2D gels were western blotted onto PVDF membrane (Roche). Blots were incubated in PBS containing 10% blocking reagent (Roche) for 30 min at room temperature, and then with patient sera at dilutions of between 1∶100 and 1∶500. Blots were washed three times for 10 min each with PBS containing 0.1% Tween® 20 (PBS-Tw), and then incubated with 1∶10,000 dilution of anti-human IgG conjugated with peroxidase (Sigma) for 30 min at room temperature followed by three 10 min washes with PBS-Tw. Blots were developed with CSPD (Roche) and exposed.

Developed films were overlain with replica silver stained 2D gels and spots that aligned with spots on the western blot were determined. These immunogenic spots were picked and washed twice for 20 min each with 50% acetonitrile (ACN), 50% 400 mM NH_4_HCO_3_ (ABC). Gel plugs were left in 100% ACN for 10 min and then air dried. The plugs were incubated in 10 mM DTT in 50 mM ABC for 30 min at 60°C followed by 30 min at room temperature in the dark with 100 mM IAA in 100 mM ABC. The gel plugs were again washed twice in 50% ACN, 50% 400 mM ABC and again incubated for 10 min in 100% ACN and air dried. The pellet was digested for 3 h with 50 ng trypsin (Promega) in 5 µl 10 mM ABC.

### Mass spectrometry

Proteins were identified by Matrix-assisted laser desorption time of flight mass spectrometry (MALDI-TOF) using a Bruker Autoflex® machine. Matrix solutions were freshly prepared by dissolving α-cyano-α-hydroxycinnamic acid (α-CCA) in acetone to saturation and spotted onto either a stainless steel or AnchorChip™ target plates and allowed to dry. A 0.5 µl drop of trypsin digested sample was placed on the matrix and the peptides allowed to adsorb for 1–2 min before being removed and the samples washed with 10% formic acid. Mass spectra were acquired with between 300–1200 laser pulses in positive reflective mode with an acceleration voltage of 19 kV. Samples were calibrated with Peptide Calibration Standard (Bruker). The peptide masses were obtained from the spectra using XMASS 5.1 (Bruker) and used to search the neisserial proteins in the NCBI database using MASCOT (Matrix Science) with search criteria of 50 ppm error, fixed modifications of carbamidomethyl modified cysteine residues, variable modifications of oxidised methionines and allowing 1 missed trypsin cleavage. Protein identities were considered reliable if the expected probability was <0.05 (details of methods are available in Document S1 and Table S2).

### Cloning and expression

Proteins that had been identified by MALDI analysis were cloned and expressed using the pET101-TOPO system (Invitrogen). Primers for the N and C terminals of the annotated ORFs from *N. meningitidis* MC58 genome (http://cmr.tigr.org) were used to PCR amplify products from L91543 genomic DNA prepared according to Bjorvatin *et al.* [53]. PCR reactions contained 10 mM Tris pH 8.8, 25 mM KCl, 5 mM (NH_4_)_2_SO_4_, 2 mM MgSO_4_, 0.2 µM primers, 0.02 U µl^−1^ *Pwo* (Roche) and 0.2 mM dNTPs. Cycle conditions were 94°C for 5 min, followed by 35 cycles of 94°C for 30 sec, 58°C for 30 sec and 72°C for 2 min, followed by 5 min at 72°C. Products were mixed with pET101-TOPO vector and transformed into TOP10 cells (Invitrogen). The correct constructs were identified by PCR and restriction digests, and the plasmids transformed in BL21 cells. The pET21b plasmid expressing factor H binding protein (fHBP), NMB1870 was kindly supplied by Chris Tang (Imperial College, London). Proteins were expressed by growing BL21 transformants to approximately OD_600_ 0.6 and inducing with IPTG to 1 mM. Cells were incubated for 3 h, the cultures centrifuged and the pellet lysed by resuspending in BugBuster® (Novagen) with 2 µg ml^−1^ DNase and 50 µg ml^−1^ lysozyme for 20 min. Lysates were centrifuged and the cleared supernatant applied to a previously equilibrated HisSelect™ (Sigma) column. The column was washed with 50 mM NaPO_4_, pH 8, 0.3 M NaCl, 5 mM imadazole and the proteins eluted with 50 mM NaPO_4_, pH 8, 0.3 M NaCl, 250 mM imadazole. Proteins were dialysed against 1000× volumes of PBS and quantified using a 2D Quant kit (Amersham).

### Mouse Immunizations

Recombinant proteins were used to generate serum according to Giuliani *et al.* [5]. Briefly, female, NIH mice were immunised subcutaneously on days 0, 21 and 35 with 20 µg protein each, initially with Freund's complete adjuvant and subsequently with Freund's incomplete adjuvant. Blood was collected on day 49 and the serum frozen. Animals were housed and cared for according to UK Home Office codes of practice and experiments were approved by the University of Surrey Ethics Committee.

### ELISAs

For ELISAs with recombinant protein, 50 µl of 2 µg ml^−1^ was added to each Maxisorp™ (Nunc) microtiter well and incubated overnight at 4°C. For whole cell ELISAs, bacteria were grown for 4 h on CAB, cells were resuspended in PBS and the OD_600_ adjusted to 0.05. Cells were heated at 56°C for 30 min, followed by 5 min of sonication and 50 µl added to Maxisorp™ ELISA wells and dried. All plates were washed three times in PBS-Tw and blocked for 90 min at 37°C with 50 µl PBS containing 10% blocking agent (Roche). A serial dilution of serum in PBS with 10% blocking agent was added to the wells and the plates incubated for 2 h at 37°C. Plates were washed three times with PBS-Tw and anti-mouse IgG peroxidase conjugate (Sigma) diluted 1∶10,000 in PBS with 10% blocking agent added. Plates were incubated for 1 h in the dark at 37°C and washed three times in PBS-Tw. Colour reagent, 3,3′,5,5′-Tetramethylbenzidine (TMB) was dissolved in 0.05 M phosphate-citrate buffer, pH 5 containing 10% DMSO and added to each well, the colour was allowed to develop and the reaction stopped by adding H_2_SO_4_ to 0.4 M. The OD_450_ of each well was measured. Titres were determined as the last dilution at which the OD_450_ was greater than 0.1 and more than twice the value for the equivalent dilution containing negative sera (from mice immunised with PBS alone).

### Serum Bactericidal Assays

Serum Bactericidal Assays (SBAs) were carried out according to Borrow and Carlone [54]. Briefly, *N. meningitidis*, L91543, grown overnight on CAB were inoculated into Mueller Hinton broth to an OD_600_ 0.1, and shaken at 35°C for 4 h. Cultures were diluted to OD_600_ 0.1 and then diluted a further 1∶5,000, 10 µl was added to microtiter wells containing a 10 µl of a 1∶4 dilution of baby rabbit complement (Serotech, UK) and 20 µl of a twofold serial dilution of heat denatured (56°C for 30 min) sera. Plates were incubated for 1 h and samples plated onto CAB media. Each assay included control wells with either no sera, heat inactivated complement, or neither sera nor active complement. Positive wells were those containing 50% of the number of cells in the control wells. SBA results for recombinant proteins were compared to sera from mice immunised with PBS alone, OMVs (as a positive control for the SBA), and the known bactericidal antigen fHBP (assayed with *N. meningitidis* MC58, as a positive control for the recombinant protein production and immunization protocols)

### Flow Cytometry

*N. meningitidis* for flow cytometry were grown for 4 h on CAB, resuspended in PBS and adjusted to an OD_600_ of 1. A 20 µl volume of cells was centrifuged, the pellet resuspended in 10 µl heat denatured sera and incubated for 30 min at 37°C. The cells were washed three times in 100 µl PBS-Tw and resuspended in 100 µl 4% paraformaldehyde for 18 h at 4°C. Cells were washed three time in 100 µl PBS-Tw and resuspended in 20 µl anti-mouse IgG conjugated to FITC (Sigma) diluted 1∶50 in PBS. Cells were incubated for 30 min at room temperature in the dark, washed three times in PBS-Tw and resuspended in 1 ml PBS. Fluorescence was measured with a BD FACSCanto™ Flow Cytometer recording 10,000 events and the data compared to samples containing either no cells, or cells stained with sera from mice immunised with fHBP, OMV preparations or PBS alone. Data were analysed using WinMDI, recorded events were gated with reference to the control samples and the population statistics determined from triplicate experiments.

## Supporting Information

Table S1 Mass Spectrometry Data
(0.05 MB XLS)

Table S2 MS exclusion list
(0.03 MB XLS)

Document S1 Parameters for MS data acquisition and database searching
(0.03 MB DOC)
