# Supplementary material for: Towards the Immunoproteome of Neisseria meningitidis
Source: PLoS One. 2009 Jun 16;4(6):e5940. doi: 10.1371/journal.pone.0005940 (PMC2691954; doi:10.1371/journal.pone.0005940)
Supplement: Document S1 — Parameters for MS data acquisition and database searching (0.03 MB DOC) [file pone.0005940.s003.doc]

**Document S1 - Parameters for MS data acquisition and database searching**

Peak List were generated by XMASS5.1, version 1.0 (Bruker Daltonik, Germany) using default parameters. Masses indicative of the matrix, trypsin and keratin, the three most common contaminants, were removed according to the exclusion list. Calibration was carried on the MALDI-TOF after every 3^rd^ sample using Bruker Peptide Calibration Standard.

Peak Lists were submitted to be searched against trypsin digests (1 missed cleavage was permitted) of all *Neisseria meningitidis* entries of the NCBI database using the public available MASCOT server (<http://www.matrixscience.com/search_form_select.html>) during 2005 and 2006 by Biotools v2.1. Carbamidomethyl (C) was used as a fixed modification and Oxidation (M) as a variable modification, no protein mass was given, mass values were for MH^+^ ions, a peptide tolerance of 50 ppm was used with monoisotopic values.

Identifications were considered significant if they had Mowse score of >50 and an expected probability of p<0.05.
